# Interferon-β Signaling Contributes to Ras Transformation

**DOI:** 10.1371/journal.pone.0024291

**Published:** 2011-08-29

**Authors:** Yu-Chen Tsai, Sidney Pestka, Lu-Hai Wang, Loren W. Runnels, Shan Wan, Yi Lisa Lyu, Leroy F. Liu

**Affiliations:** 1 Department of Pharmacology, University of Medicine and Dentistry of New Jersey-Robert Wood Johnson Medical School, Piscataway, New Jersey, United States of America; 2 Department of Molecular Genetics, Microbiology and Immunology, University of Medicine and Dentistry of New Jersey-Robert Wood Johnson Medical School, Piscataway, New Jersey, United States of America; 3 Division of Molecular and Genomic Medicine, National Health Research Institutes, Miaoli County, Taiwan; Yale Medical School, United States of America

## Abstract

Increasing evidence has pointed to activated type I interferon signaling in tumors. However, the molecular basis for such activation and its role in tumorigenesis remain unclear. In the current studies, we report that activation of type I interferon (IFN) signaling in tumor cells is primarily due to elevated secretion of the type I interferon, IFN-β. Studies in oncogene-transformed cells suggest that oncogenes such as Ras and Src can activate IFN-β signaling. Significantly, elevated IFN-β signaling in Ras-transformed mammary epithelial MCF-10A cells was shown to contribute to Ras transformation as evidenced by morphological changes, anchorage-independent growth, and migratory properties. Our results demonstrate for the first time that the type I IFN, IFN-β, contributes to Ras transformation and support the notion that oncogene-induced cytokines play important roles in oncogene transformation.

## Introduction

Increasing evidence has indicated up-regulated type I interferon (IFN) signaling in many tumors. For example, transcriptional profiling studies of invasive squamous cell carcinoma of the skin have demonstrated elevated expression of IFN-regulated genes [1]. Enhanced IFN signaling has also been suggested in a proteomic study of oral squamous cell carcinoma [2]. Most notably, ISG15 (interferon-stimulated gene 15), has been shown to be a new tumor marker for many cancers [3]. ISG15, whose expression is controlled by type I interferons, is an ubiquitin-like protein (UBL). Unlike ubiquitin whose expression is more or less constant in all cells, ISG15, which is undetectable in most normal tissues [4], is highly expressed, albeit with a high degree of heterogeneity, in both tumor cell lines and tumor biopsies. For example, among a panel of breast cancer cell lines, ISG15 is highly expressed in ZR-75-1 and MDA-MB-231, but not in BT-474 cells [4]. In addition, studies of biopsy samples have demonstrated that ISG15 is highly elevated and variably expressed in endometrium tumors, but non-detectable in their normal counterpart tissues [4]. Similar analysis has also revealed highly elevated expression of ISG15 in bladder, prostate and oral cancers [2], [5], [6]. Transcriptomic dissection of the head and neck/oral squamous cell carcinoma (HNOSCC) has also identified the ISG15 gene as an up-regulated gene [7]. Furthermore, elevated ISG15 expression in bladder cancers shows a positive correlation with the stages of the disease [5]. Significantly, ISG15 has been shown to be a prognostic marker for breast cancer [8].

Elevated ISG15 expression in various tumors suggests up-regulation of type I IFN signaling in these tumors. However, the molecular basis for ISG15 overexpression and activated IFN signaling in tumors remains unclear. One possibility is that elevated expression of ISG15 and hence increased IFN signaling in tumors is linked to oncogene activation. Oncogenes, such as Ras, are known to cause cellular transformation (e.g. morphological changes and anchorage-independent growth) and play a key role in tumorigenesis [9]. More recently, the involvement of Ras in cancer invasion and metastasis has also been suggested through studies of oncogene-induced epithelial-mesenchymal transition (oncogenic EMT) in several model systems [10], [11]. Tumor cells also appear to acquire EMT characteristics during tumor invasion and metastasis, and many EMT markers such as Snail (a transcription factor) and E-cadherin are known to be dysregulated in metastatic tumors [12], [13], [14], [15].

In the current studies, we show that oncogenic Ras induces elevated ISG15 expression in human mammary epithelial MCF-10A cells due to IFN-β signaling. Furthermore, we show that IFN-β signaling through ISG15 contributes positively to Ras transformation and oncogenic EMT in MCF-10A cells, supporting the notion that oncogene-induced cytokines play important roles in oncogene transformation.

## Results

### ISG15 overexpression in breast cancer ZR-75-1 cells is due to elevated IFN-β signaling

**Figure 1:**
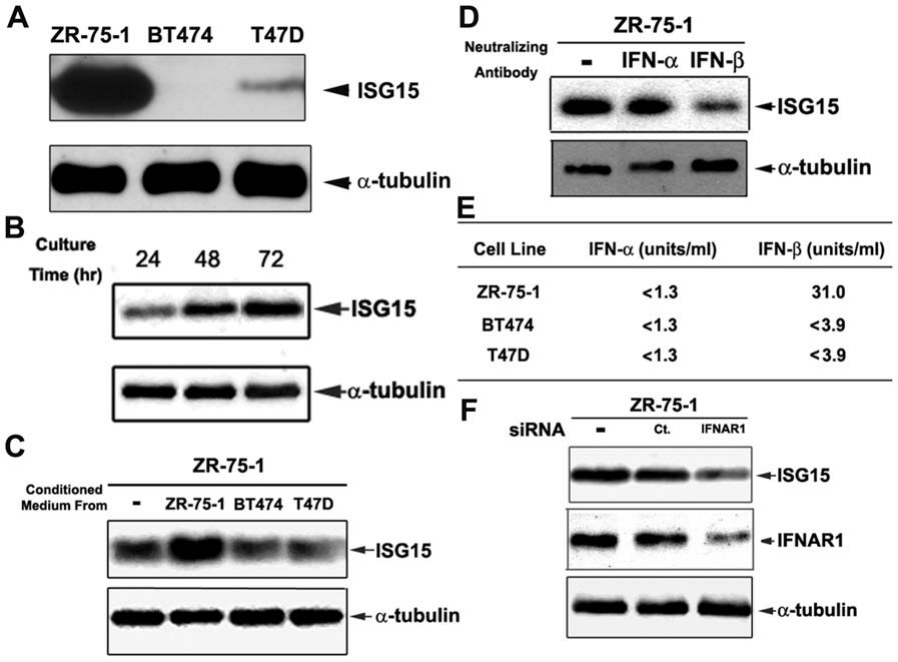
Elevated ISG15 expression in breast cancer cells is due to interferon-β (IFN-β) secretion. Breast cancer ZR-75-1, BT474, and T47D cells were used in the current studies. A. ISG15 is overexpressed in ZR-75-1 cells as compared to BT474 and T47 D cells. Steady-state levels of ISG15 were measured by immunoblotting using anti-ISG15 antibodies. B. Time-dependent increase in ISG15 expression in cultured ZR-75-1 cells. Breast cancer ZR-75-1 cells were harvested at different time intervals after seeding, and cell lysates were analyzed by immunoblotting with anti-ISG15 antibodies. C. Conditioned media from cultured ZR-75-1 cells stimulate ISG15 expression. Breast cancer ZR-75-1, BT474, and T47D cells were seeded at equal density and cultured for 3 days. Conditioned media were collected individually and filtered through 0.2 µm Corning syringe filters. The newly (16 hrs) seeded ZR-75-1 cells were then replenished with the conditioned media. Lysates were collected 3 days later and analyzed by immunoblotting with anti-ISG15 antibodies. D. Antibody neutralization of IFN-β inhibits ISG15 expression in tumor cells. ZR-75-1 cells were cultured for 3 days in the presence of neutralizing antibodies against human IFN-α or human IFN-β (100 NU/ml). Cell lysates were immunoblotted with anti-ISG15 antibodies. E. Elevated IFN-β is present in the conditioned medium from ZR-75-1 cells. Conditioned media from different breast cancer cells, ZR-75-1, BT474, and T47D were individually collected after 3 days of culturing. The media were assayed for IFN-α and IFN-β using the interferon antiviral assay as described in Materials and Methods. F. The interferon receptor is required for elevated ISG15 expression in ZR-75-1 cells. Breast cancer ZR-75-1 cells were transiently transfected with control or IFNAR1 siRNA. Cell lysates were collected 72 hrs post-transfection and immunoblotted with anti-ISG15 antibodies.

Previous studies have demonstrated that ISG15 is highly, but variably, overexpressed in tumor tissues and tumor cell lines [3], [4]. As shown in Fig. 1A, ISG15 is variably expressed in three breast cancer cell lines, with ISG15 expression being the highest in ZR-75-1 as compared to BT474 and T47D. The following observations suggest that ISG15 overexpression in ZR-75-1 cells is due to elevated interferon-β signaling: (1) We found that the ISG15 level (normalized to α-tubulin) in breast cancer ZR-75-1 cells increased with increasing culturing time (Fig. 1B). The possibility that a cytokine is involved is further suggested from the experiment that the conditioned media from (three-day) cultured ZR-75-1 cells, but not BT474 or T47D cells, were able to elevate ISG15 expression in freshly seeded ZR-75-1 cells (Fig. 1C). (2) Culturing of ZR-75-1 cells in the presence of IFN-β-, but not IFN-α-, neutralizing antibodies strongly reduced ISG15 expression (Fig. 1D), suggesting that IFN-β is secreted by ZR-75-1 cells and is responsible for elevated ISG15 expression. This notion was further supported by an interferon antiviral assay which showed the presence of IFN-β, but not IFN-α, in the ZR-75-1 cell-conditioned medium (Fig. 1E). (3) siRNA-mediated knockdown of IFNAR1 [interferon (alpha, beta and omega) receptor 1], one of the two chains of the type I IFN receptor, in ZR-75-1 cells resulted in a significant reduction of ISG15 expression (Fig. 1F), suggesting that interferon signaling through the receptor is involved in ISG15 overexpression in ZR-75-1 cells. In the aggregate, these results suggest that ISG15 overexpression in ZR-75-1 breast cancer cells is due to elevated IFN-β signaling.

### Src and Ras oncogenes stimulate ISG15 expression in cultured mammalian cells

**Figure 2:**
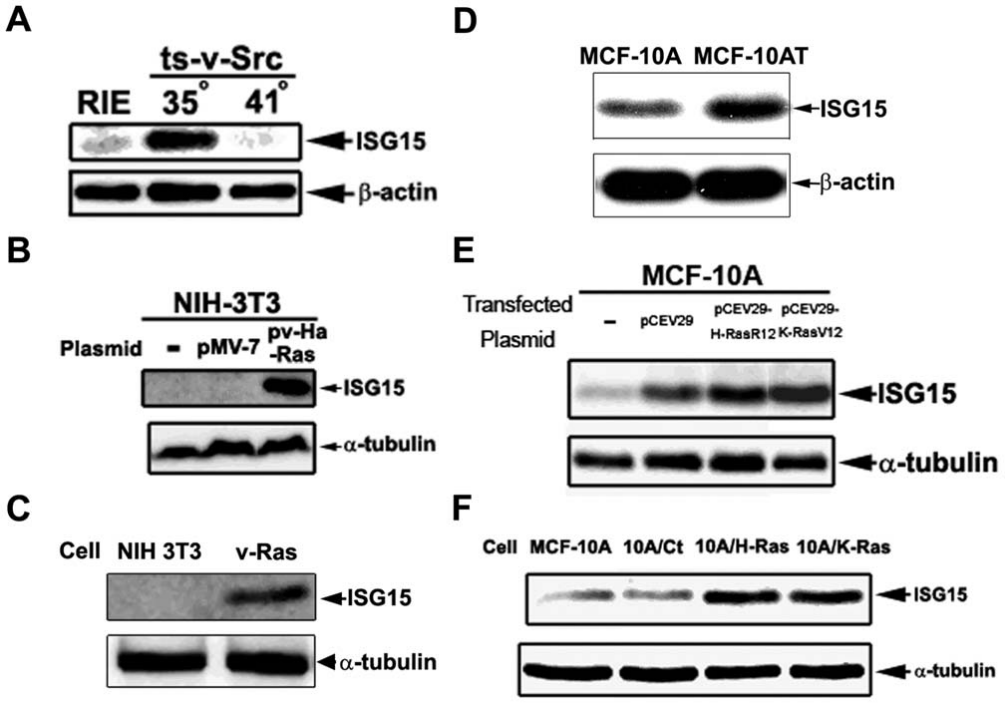
Oncogenic Ras elevates ISG15 expression. A. v-Src induces ISG15 expression in rat intestinal epithelial (RIE) cells. The RIE cell line and its ts-v-Src-transformed variant were cultured at different temperatures (35°C and 41°C) for 3 days. Lysates were analyzed by immunoblotting using anti-ISG15 antibodies. B. Oncogenic Ras induces ISG15 expression in transiently transfected NIH 3T3 cells. Murine fibroblast NIH 3T3 cells were transiently transfected with the vector or v-Ha-Ras plasmid using Polyfect (Qiagen) following the manufacturer's instructions. Cell lysates were collected 2 days post-transfection and immunoblotted with anti-ISG15 antibodies. C. Elevated ISG15 expression in oncogenic Ras-transformed NIH 3T3 cells. Oncogenic v-Ha-Ras-transformed and control NIH 3T3 cells were cultured for 2 days. Cell lysates were immunoblotted with anti-ISG15 antibodies. D. Elevated ISG15 expression in the H-Ras transformed MCF-10AT cell line. Cells were seeded equally and harvested 2 days later. Lysates were immunoblotted with anti-ISG15 antibodies. E. Transient transfection with Ras oncogenes (H-RasR12 and K-RasV12) induces ISG15 in mammary epithelial cells. Human mammary epithelial MCF-10A cells were transiently transfected with oncogenic Ras-expressing plasmids using FuGene (Roche) as described in Materials and Methods. Two days post-transfection, cell lysates were immunoblotted with anti-ISG15 antibodies. F. Oncogenic Ras-transformed mammary epithelial MCF10A cells overexpress ISG15. The oncogenic Ras-transformed MCF-10A (10A/H-Ras and 10A/K-Ras) were established as described in Materials and Methods. Culturing conditions and Western blotting were performed as described in D.

In order to determine the molecular basis for ISG15 up-regulation in tumor cells, we have evaluated the effect of oncogenes on ISG15 expression in cultured mammalian cells. Temperature-sensitive viral Src (ts-v-Src)-transformed rat intestinal epithelial (RIE) cells were used to determine the effect of the Src oncogene on ISG15 expression. At the permissive temperature (35°C), ts-v-Src cells, which are known to maintain its Src kinase activity and transformation phenotype [16], expressed a high level of ISG15 as compared to untransformed RIE cells (Fig. 2A). In addition, at the non-permissive temperature (41°C), ts-v-Src cells expressed a lower level of ISG15, similar to that of the untransformed RIE cells (Fig. 2A). These results suggest that ISG15 overexpression could be linked to oncogene activation.

To further test whether Ras may up-regulate ISG15, NIH 3T3 cells were transiently transfected with either the control vector (pMV-7) or the v-Ha-Ras expression vector (pv-Ha-Ras). As shown in Fig. 2B, ISG15 is greatly elevated in v-Ha-Ras-transfected NIH 3T3 cells as compared to vector-transfected control cells. In support of the transient transfection results, NIH 3T3 cells stably transfected with v-Ha-Ras (v-Ras) expressed a much higher level of ISG15 as compared to NIH 3T3 control cells (Fig. 2C). Furthermore, a similar study was performed using human mammary epithelial MCF-10A cells. As shown in Fig. 2E, MCF-10A cells transiently transfected with either *H-RasR12* (expressing activated H-Ras) or *K-RasV12* (expressing activated K-Ras) expressed higher ISG15 levels as compared to MCF-10A cells transfected with the control vector (pCEV29). Consistent with the transient transfection results, MCF-10A cells stably transfected with either *H-RasR12* (designated 10A/H-Ras cells) or *K-RasV12* (designated 10A/K-Ras cells) expressed much higher levels of ISG15 as compared to MCF-10A cells stably transfected with the control vector pCEV29 (designated 10A/Ct) (Fig. 2F). In addition, elevated ISG15 expression was also demonstrated in a well characterized H-Ras-transformed MCF-10A (MCF-10AT) cell line [17] as compared to its untransformed control line (MCF-10A) (Fig. 2D). Taken together, these results strongly suggest that oncogenic Ras stimulates ISG15 expression.

### Oncogenic Ras-induced ISG15 overexpression requires IFN-β signaling

**Figure 3:**
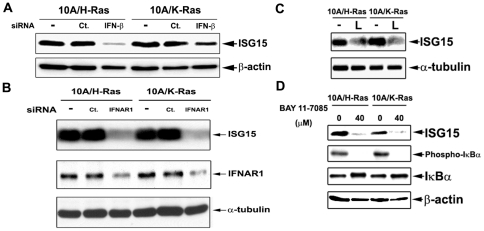
Identification of the effectors for ISG15 induction in the Ras-dependent signaling pathway. A, B. siRNA knock-down of interferon-β and the type I IFN receptor reduces ISG15 induction in the Ras-dependent signaling pathway. The oncogenic Ras-transformed MCF-10A cells (10A/H-Ras and 10A/K-Ras) were transiently transfected with control, IFN-β or IFNAR1 siRNA. Cell lysates were collected 72 hrs post-transfection and immunoblotted with anti-ISG15 and anti-IFNAR1 antibodies. C. The PI3K inhibitor reduces ISG15 expression in oncogenic Ras-transformed cells. 10A/H-Ras and 10A/K-Ras cells were treated with 20 µM LY294002, a PI3K inhibitor, for 3 days. Lysates with equal protein amount were immunoblotted with anti-ISG15 antibodies (L: LY294002). D. The NF-κB inhibitor reduces ISG15 expression in oncogenic Ras-transformed cells. 10A/H-Ras and 10A/K-Ras cells were seeded and cultured overnight. Cells were then treated with or without 40 µM Bay 11-7085, an NF-κB inhibitor, for 24 hrs. Cell lysates were analyzed by immunoblotting using anti-ISG15, phospho-IκBα, and IκBα antibodies.

As demonstrated in Fig. 1, overexpression of ISG15 in ZR-75-1 cells is due to elevated IFN-β secretion and activation of the type I IFN signaling. To determine whether Ras-induced ISG15 overexpression is also due to elevated IFN-β signaling, IFN-β and IFNAR1 were silenced by their specific siRNAs in Ras-transformed MCF-10A cells (10A/H-Ras and 10A/K-Ras). As shown in Fig. 3, knocking down either IFN-β (Fig. 3A) or IFNAR1 (Fig. 3B) in oncogenic Ras-(H-Ras- or K-Ras-) transformed cells resulted in decreased expression of ISG15. These results suggest that elevated IFN-β signaling is responsible for ISG15 overexpression in oncogenic Ras-transformed MCF-10A cells.

In addition to the involvement of IFN-β and IFNAR1, the Ras downstream signals necessary for ISG15 overexpression was also investigated using specific inhibitors. Oncogenic Ras-transformed MCF-10A cells were treated with LY294002, a PI3K inhibitor. Interestingly, the ISG15 level was reduced by the PI3K inhibitor (labeled L) in both Ras-transformed lines (Fig. 3C), suggesting that the PI3K signaling pathway is involved in ISG15 induction in Ras-transformed MCF-10A cells. Since NF-κB is known to regulate IFN-β expression through its binding to the IFN-β promoter, we also investigated the possible involvement of NF-κB in Ras-induced ISG15 overexpression [18]. As shown in Fig. 3D, treatment of both H- and K-Ras-transformed MCF-10A cells with the NF-κB inhibitor, Bay 11-7085, resulted in significant reduction of the ISG15 level, suggesting a possible involvement of NF-κB in Ras-induced ISG15 overexpression in MCF-10A cells. Together, these results suggest that PI3K and NF-κB are potential Ras downstream signals necessary for ISG15 overexpression in Ras-transformed MCF-10A cells.

### IFN-β signaling modulates Ras transformation in MCF-10A cells

**Figure 4:**
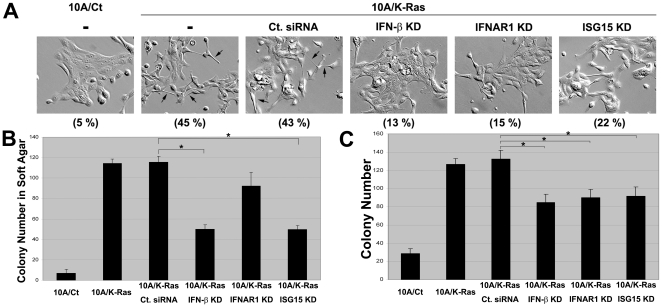
IFN-β autocrine signaling through ISG15 contributes to Ras transformation. A. Oncogenic Ras-induced morphological changes are reversed by siRNA-mediated knockdown of IFN-β, IFNAR1 or ISG15. 10A/K-Ras cells were transiently transfected with control, IFN-β, IFNAR1 or ISG15 siRNA. Images of cell morphology were taken three days later. Some of the cells with spindle-shaped morphology are indicated by arrows in 10A/K-ras cells treated with (third panel) and without (second panel) control siRNA (Ct. siRNA). Percentages of spindle-shaped cells (based on counting of 50 cells per field in three randomly selected fields) are shown at the bottom of each panel in parenthesis. B. Ras-induced anchorage-independent growth is reduced by knocking down IFN-β, IFNAR1 or ISG15. 10A/K-Ras cells were transiently transfected with different siRNAs. Three days later, cells were used to perform soft agar colony formation assay as described in Materials and Methods. Results (number of colonies in soft agar/well) were averages of triplicates. C. Ras-enhanced colony formation is reversed by knocking down IFN-β, IFNAR1, or ISG15. 10A/K-Ras cells were transiently transfected with different siRNAs. Three days post-transfection, untreated and transfected cells were trypsinized, counted and seeded in 35 mm plates (500 cells/plate) in triplicates per treatment. After culturing for 10 days, colonies were stained with methylene blue and counted. Results (number of colonies/plate) were averages of triplicates (* indicates p-value <0.05). All experiments have been repeated once with similar results.

Ras transformation is known to result in major cell morphology changes and anchorage-independent growth [9]. To investigate a possible role of IFN-β signaling in Ras transformation, we transiently knocked down IFN-β, IFNAR1 or ISG15, and monitored cellular morphology and colony formation (in both attached and suspended conditions) in K-Ras-transformed MCF-10A cells (10A/K-Ras). As shown in Fig. 4A, the percentage of spindle-shaped cells in 10A/K-Ras population was around 45% as compared to 5% in 10A/Ct population. The percentage of spindle-shaped cells was reduced to 13, 15, and 22% when IFN-β, IFNAR1 and ISG15 were knocked down by their respective siRNAs in 10A/K-Ras cells. By contrast, the percentage of spindle-shaped cells of control siRNA-transfected 10A/K-Ras cells was nearly unchanged (43%), suggesting that the cell morphology change upon Ras transformation is dependent on IFN-β, IFNAR1 and ISG15. To determine if IFN-β signaling may contribute to anchorage-independent growth in Ras-transformed MCF-10A cells, soft agar colony formation assay was performed. As shown in Fig. 4B, 10A/K-Ras cells formed about 11 times more colonies in soft agar than 10A/Ct cells. However, siRNA-mediated knockdown of IFN-β, IFNAR1 or ISG15 reduced the colony formation efficiency of 10A/K-Ras cells to 50-70% (Fig. 4B). We also performed the colony formation assay on plastic surfaces. As shown in Fig. 4C, sparsely seeded 10A/K-Ras cells clearly formed more colonies than 10A/Ct cells (about 4-fold difference). The colony formation efficiency was reduced to about 70% when IFN-β, IFNAR1 or ISG15 was knocked down in 10A/K-Ras cells. All results are statistically significant (p-value <0.05, marked by *) with the possible exception of the IFNAR1 knockdown result (p-value = 0.07) in soft agar assay (Fig. 4B). These results suggest that IFN-β signaling contributes significantly to Ras-transformation in MCF-10A cells.

### IFN-β signaling contributes to migratory properties of Ras-transformed MCF-10A cells

**Figure 5:**
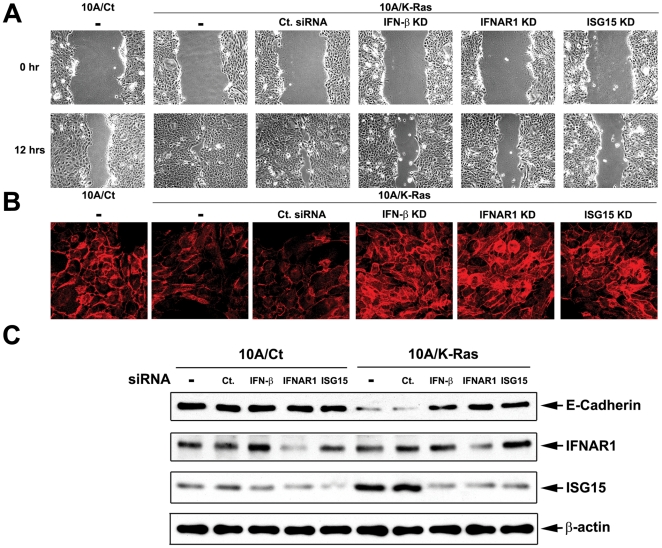
IFN-β autocrine signaling through ISG15 contributes to migratory properties of Ras-transformed MCF-10A cells. A. Oncogenic Ras-elevated cell motility is reversed by knocking down IFN-β, IFNAR1, or ISG15. 10A/K-Ras cells were transiently transfected with different siRNA. 24 hrs post-transfection, cells were trypsinized, seeded and cultured overnight to reach 100% confluence, followed by assaying wound healing efficiency as described in Materials and Methods. The percentage of wound area in the viewing fields from 3 different images per treatment is summarized. B. Cellular E-cadherin expression is restored by knocking down IFN-β, IFNAR1 or ISG15 in Ras-transformed cells. 10A/K-Ras cells were transfected with various siRNAs. Two days post-transfection, cells were trypsinized and seeded onto cover slips pre-coated with poly-L-Lysine and fibronectin. After culturing for one day, cells were processed for E-cadherin staining (in red) as described in Materials and Methods. C. IFN-β autocrine signaling through ISG15 modulates E-cadherin in Ras-transformed cells. 10A/Ct and 10A/K-Ras cells were transfected with various siRNAs for 3 days. Cell lysates were immunoblotted with various antibodies.

Ras is known to confer increased cell migration and oncogenic epithelial-mesenchymal transition (EMT), which have been linked to tumor invasion and metastasis [10]. In order to determine a possible role of IFN-β signaling in oncogenic EMT, we have monitored the effect of IFN-β signaling on increased cell migration and appearance of the EMT marker E-cadherin in 10A/K-Ras cells. Cell migration was assessed by a wound healing assay (see Materials and Methods). As shown in Fig. 5A, the wound area of 10A/Ct cells was reduced to about 40% 12 hrs post-wounding. By contrast, the wound area of 10A/K-Ras was nearly completely closed (reduced to about 6%), suggesting that 10A/K-Ras cells migrate faster than 10A/Ct cells. When IFN-β, IFNAR1 or ISG15 was knocked down by their respective siRNA in 10A/K-Ras cells, the wound area was reduced to the level of the control 10A/Ct cells (about 50%), suggesting that IFN-β signaling contributes positively to increased cell migration in Ras-transformed cells.

We have also assessed the effect of IFN-β signaling on the appearance of the key EMT marker E-cadherin in Ras-transformed MCF-10A cells [12], [19]. As shown in Fig. 5C, E-cadherin was shown to be down-regulated in 10A/K-Ras cells and silencing of IFN-β, IFNAR1 or ISG15 resulted in significant restoration of the E-cadherin protein level as evidenced by both immunoblotting (Fig. 5C) and immunofluorescence (Fig. 5B, note the red E-cadherin staining on cellular surfaces) using anti-E-cadherin antibodies. In the aggregate, these results suggest that IFN-β signaling through ISG15 contributes to the migratory properties of Ras-transformed MCF-10A cells.

## Discussion

The demonstration of ISG15 as a tumor marker immediately suggests that the type I IFN signaling pathway may be up-regulated in tumors. Indeed, transcription profiling studies have demonstrated up-regulation of type I IFN-regulated genes in some tumors [1]. Our current studies have suggested that the increased secretion of IFN-β with subsequent activation of the type I IFN signaling pathway is responsible for ISG15 overexpression in breast cancer ZR-75-1 cells. We have also asked the question whether the elevated ISG15 expression in tumor cells could be due to oncogene activation. Indeed, we have shown that both Src and Ras can stimulate ISG15 expression in tissue culture cells. Using oncogenic Ras-transformed human mammary epithelial MCF-10A cells, we have further demonstrated that oncogenic Ras up-regulates ISG15 due to activation of IFN-β signaling through the type I IFN receptor, IFNAR. In addition to Src and Ras as demonstrated in the current studies, E1A has previously been shown to cause elevated expression of IFN-β and ISG15 in the adenovirus type 5 (Ad5)-transformed rodent cells [20]. Taken together, these results suggest that oncogenes can up-regulate ISG15 through elevated expression of IFN-β.

In our current studies, we have demonstrated for the first time that IFN-β signaling through ISG15 contributes positively to oncogenic transformation (e.g. changes in cell morphology and anchorage-independent growth) in Ras-transformed MCF-10A cells. Our studies have also demonstrated that elevated IFN-β signaling through ISG15 contributes significantly to cell morphology changes, cell migration properties and reduced expression of the EMT marker E-cadherin. Since oncogenic EMT has been suggested to play an important role in tumor invasion and metastasis [10], our results could implicate a role of IFN-β signaling in tumor progression.

In our studies, oncogenic Ras appears to stimulate IFN-β expression and secretion through activation of the PI3K/NF-κB pathway. PI3K, which can be directly activated by Ras, is known to control IRF-3, an important transcription factor regulating IFN-β expression [21]. However, PI3K is also known to activate NF-κB [22], a transcription factor which is found active in many types of tumors and associated with tumor phenotypes [23]. Among different type I IFN genes, the NF-κB binding site is found on the IFN-β, but not the IFN-α, promoter [24]. Our results thus suggest that active NF-κB may be an important regulator in this oncogene-induced IFN-β signaling. In this regard, it is interesting to point out that oncogenic Ras has been shown to induce IL-6 and IL-8, both of which are NF-κB-regulated genes and shown to promote Ras transformation and tumor growth [25], [26], [27]. Interestingly, the type I IFN, IFN-α, has been shown to induce IL-6 in myeloma cells [28]. It seems likely that the complex interplay among various oncogene-induced cytokines is critical for oncogene transformation and tumorigenesis.

Our results, which demonstrate a positive role of type I IFN signaling in oncogene transformation and oncogenic EMT, could suggest therapeutic strategies for cancer treatment by antagonizing the type I IFN signaling pathway in tumor cells. However, type I IFNs are known to suppress tumor growth and IFN-α (i.e. IFN-α2) has been used for the treatment of several cancers [29]. Several possibilities could explain this apparent paradox: (1) The effect of type I IFNs on tumor cell growth could be concentration-dependent. At high concentrations such as those used in IFN therapy, type I IFNs could suppress tumor growth through stimulation of complex immune responses against the tumors (e.g. up-regulation of tumor antigen presentation through MHC class I molecules) and inhibition of tumor cell proliferation [29]. At lower concentrations, IFNs may contrastingly stimulate tumor growth [30]. Indeed, only low concentrations of type I IFNs have been shown to stimulate the clonogenic tumor growth *in vitro* in a fraction (6-28%) of human tumor samples tested [30]. Our current results, which demonstrate a positive role of the IFN-β signaling in oncogenic transformation and EMT, are consistent with the growth stimulatory effect of IFNs at lower concentrations. (2) IFN-α and IFN-β may exhibit different or opposite effects on tumor cell growth, despite the fact that they share the same receptor for signaling. Indeed, it has been reported that the gene expression patterns induced by low concentrations of IFN-α and IFN-β overlap but are not identical [31]. In addition, while IFN-α is used for cancer therapy, IFN-β is used for treatment of multiple sclerosis [32]. (3) The effect of type I IFNs on tumor cell growth could be dictated by the genetic makeup of the tumor cells. It is well known that tumor cells can develop various immune escape mechanisms through mutations, including MHC class I deficiencies, to escape from IFN-mediated immune suppression [33]. For example, MHC class I deficiencies (e.g. mutations of the HLA genes and defects in the components of the antigen processing machinery) occur at near 100% frequency in some types of tumors [33]. It seems possible that tumor cells may also adopt different mechanisms to escape the growth inhibitory activities of type I IFNs (e.g. BT474 cells, which do not express detectable ISG15, could have inactivated the IFN signaling pathway through mutations to escape either the immune suppressive or the growth-inhibitory activities of IFNs). Consequently, many tumors may have escaped the growth suppressive activities of type I IFNs, but retain their dependence on type I IFNs for anchorage-independent growth and metastasis. It is also important to point out that the activities of type I IFN signaling may also involve modulations by the complex cytokine network in the tumor microenvironment. Clearly, further studies are necessary to clarify the role of type I IFN signaling in tumorigenesis.

## Materials and Methods

### Cells and cell culture

ZR-75-1, BT474 and T47D cells [4], [34] were cultured in RPMI supplemented with 10% Fetalplex (Gemini Bio-Products, West Sacramento, CA, USA), L-glutamine (2 mM), penicillin (100 units/ml) and streptomycin (100 µg/ml) in a 37°C incubator with 5% CO_2_. RIE, ts-v-Src transformed RIE, NIH 3T3, and v-Ras-transformed NIH 3T3 cells were cultured similarly except using DMEM instead of RPMI. MCF-10A and MCF-10AT cells were cultured in Advanced DMEM/F12 (Invitrogen, Carlsbad, CA, USA) supplemented with 5% equine serum (Invitrogen), 20 ng/ml recombinant human EGF (Invitrogen), 10 µg/ml insulin (Sigma, St. Louis, MO, USA), 0.5 µg/ml hydrocortisone (Sigma), 100 ng/ml cholera toxin (Sigma), L-glutamine (2 mM), penicillin (100 units/ml) and streptomycin (100 µg/ml) in a 37°C incubator with 5% CO_2_.

### Immunoblotting

Cells with various treatments were lysed with 6X sample buffer. After boiling for 10 min, cell lysates were analyzed by SDS-PAGE followed by immunoblotting with the appropriate antibody. The following antibodies were used: IFNAR1 (ab45172; Abcam, Cambridge, MA, USA), E-cadherin (#3195; Cell Signaling Technology), IκBα (#4814; Cell Signaling Technology), phospho-IκBα (#9246; Cell Signaling Technology) and β-actin (#4970; Cell Signaling Technology). Visualization of bands was performed using SuperSignal West Pico Chemiluminescent Substrate (Pierce, Rockford, IL, USA) and Kodak Image Station 2000R.

### Interferon antiviral assay

Antiviral assays were performed as previously described [35]. In brief, MDBK (for determining the concentration of IFN-α) or A549 cells (for determining the combined concentration of IFN-α and IFN-β) were plated in 96-well plates containing 2-fold serial-diluted laboratory standard IFN-α, or IFN-β, or conditioned media from different cancer cells. VSV (vesicular stomatitis virus) and EMCV (encephalomyocarditis virus) were later added into MDBK and A549 cells, respectively. When cells in wells containing no interferon were lysed by virus, plates were drained and stained with crystal violet to visualize live cells. Interferon concentrations were determined by comparing results to laboratory standard IFNs.

### siRNA knockdown

siRNA against IFN-β (s7189, Ambion, Austin, TX, USA), IFNAR1 (s783, Ambion), ISG15 (s228517, Ambion), and control siRNA (SIC001, Sigma) were transfected into cells using Oligofectamine transfection reagent (Invitrogen). The specific conditions for each knockdown experiment are described in the figure legends.

### Construction of transformed MCF-10A cells

MCF-10A cells were transfected with control vector (pCEV29), oncogenic H-Ras (pCEV29-H-RasR12) and oncogenic K-Ras (pCEV29-K-RasV12) expressing vectors using FuGene transfection reagent (Roche) to create 10A/Ct, 10A/H-Ras and 10A/K-Ras cells, respectively. Three days post transfection, cells were replenished with 800 µg/ml G-418 (Invitrogen)-containing fresh medium, and repeated passages were performed in the same media until cells became resistant to G-418.

### Soft agar colony formation assay

Cells (3×10^3^) were seeded in 2 ml of 0.35% agar (Sigma) supplemented with complete MCF-10A medium. This suspension was layered over 2 ml of 0.6% agar-medium base in each well of a 6-well plate. 2 ml of complete medium was added after the agar was solidified. Cells were replenished with fresh media every week for 3 weeks, followed by staining with 1 mg/ml p-Iodonitrotetrazolium violet. Colonies larger than 0.2 mm in diameter were counted using MiniCount (Imaging Products International).

### Wound healing assay

10A/Ct and 10A/K-Ras cells were transfected with various siRNAs. 24 hrs post-transfection, transfected cells were seeded at a density of 1.3×10^6^ cells/35 mm plate. After another 24 hrs, cells were replenished with 1 µg/ml mitomycin C (Sigma)-containing fresh media. The wound was created by scratching cells with pipette tips (200 µl tips). Images of wound areas were taken at 0 and 12 hr after scratching. Analysis of the wound area (expressed in percentage of the total imaged area) was performed using the ImageJ program.

### Immunofluorescence and confocal microscopy

Various MCF-10A cells were transfected with and without siRNAs (as indicated in the figures) for 48 hrs. Transfected cells were then plated onto poly-L-Lysine- and fibronectin-coated coverslips. 24 hrs after seeding, cells were fixed at room temperature for 10 min in phosphate-buffered saline (pH 7.4) with 4% paraformaldehyde (Electron Microscopy Sciences, Hatfield, PA, USA) and permeabilized in phosphate-buffered saline with 0.2% Triton X-100 (Fisher Scientific, Waltham, MA, USA) followed by treatment with 10% BSA in phosphate-buffered saline for 1 hr. Primary (rabbit) antibodies against E-cadherin were incubated with samples for 8 hrs followed by incubation with a secondary antibody (Cy3 goat antibody against rabbit IgG) (Jackson ImmunoResearch Laboratories, West Grove, PA, USA). The images were captured using a Zeiss LSM 410 confocal microscope with a 568-nm excitation wavelength and a 610-nm band pass emission filter. The pinhole size used was 30 Airy Units, and the contrast/brightness settings were kept the same for all images.
